# Supplementary material for: ProteinShader: illustrative rendering of macromolecules
Source: BMC Struct Biol. 2009 Mar 30;9:19. doi: 10.1186/1472-6807-9-19 (PMC2672931; doi:10.1186/1472-6807-9-19)
Supplement: Additional file 1 — ProteinShader program without source code. This compressed file contains the complete ProteinShader program including associated libraries, but no source code. A README.txt file gives an overview of the ProteinShader distribution, and the index.html file in the help subdirectory has directions on getting started with the program as well as a set of tutorials. [file 1472-6807-9-19-S1.zip › ProteinShader-beta-0_9_4-binary/help/api/org/proteinshader/gui/enums/class-use/ImageFormatEnum.html]

Uses of Class org.proteinshader.gui.enums.ImageFormatEnum (ProteinShader API)


|  |  |  |
| --- | --- | --- |
| **Overview** \| **Package** \| **Class** \| **Use** \| **Tree** \| **Deprecated** \| **Index** \| **Help** | |  |
| PREV   NEXT | **FRAMES**    **NO FRAMES**     **All Classes** |  |


---


## **Uses of Class org.proteinshader.gui.enums.ImageFormatEnum**

| Packages that use ImageFormatEnum | |
| --- | --- |
| **org.proteinshader.gui** | Holds all of the Swing GUI components and their associated listeners, including class Renderer, which is registered as a listener for the GLCanvas object that is used a drawing surface. |
| **org.proteinshader.gui.enums** | Holds the enums needed by org.proteinshader.gui and its subpackages. |
| **org.proteinshader.gui.exceptions** | Holds the exception classes needed by org.proteinshader.gui and its subpackages. |
| **org.proteinshader.gui.utils** | Holds minor utility classes needed by the Renderer. |
| **org.proteinshader.gui.viewing** | Holds important helper classes that the Renderer uses to control the view: Camera, Rotation, Lighting, *etc*. |

Uses of ImageFormatEnum in org.proteinshader.gui

| Methods in org.proteinshader.gui with parameters of type ImageFormatEnum | |
| --- | --- |
| `void` | `MediatorImpl.takeScreenShot(ImageFormatEnum format, File file)`             Causes the Render to take a screen shot of the current canvas image and save it as a JPEG, PNG or GIF file. |
| `void` | `Renderer.takeScreenShot(ImageFormatEnum format, File file)`             Sets up a screen shot so that the next call to display() will cause the current image on the canvas to be saved to a JPEG, PNG or GIF file. |
| `void` | `Mediator.takeScreenShot(ImageFormatEnum format, File file)`             Causes the Render to take a screen shot of the current canvas image and save it as a JPEG, PNG or GIF file. |

Uses of ImageFormatEnum in org.proteinshader.gui.enums

| Methods in org.proteinshader.gui.enums that return ImageFormatEnum | |
| --- | --- |
| `static ImageFormatEnum` | `ImageFormatEnum.valueOf(String name)`             Returns the enum constant of this type with the specified name. |
| `static ImageFormatEnum` | `ImageFormatEnum.valueOfMenuName(String menuName)`             Returns the ImageFormatEnum with the same menu name as the String given as an argument. |
| `static ImageFormatEnum[]` | `ImageFormatEnum.values()`             Returns an array containing the constants of this enum type, in the order they're declared. |

Uses of ImageFormatEnum in org.proteinshader.gui.exceptions

| Methods in org.proteinshader.gui.exceptions that return ImageFormatEnum | |
| --- | --- |
| `ImageFormatEnum` | `ScreenShotException.getFormat()`             Returns the format type for the image file that could not be written. |

Constructors in org.proteinshader.gui.exceptions with parameters of type ImageFormatEnum

`ScreenShotException(ImageFormatEnum format,
File file,
String message)`
  
          The message given this constructor can be retrieved using
getMessage().

`ScreenShotException(ImageFormatEnum format,
File file,
String message,
String lowerLevelMessage)`
  
          The message given this constructor can be retrieved using
getMessage().

Uses of ImageFormatEnum in org.proteinshader.gui.utils

Constructors in org.proteinshader.gui.utils with parameters of type ImageFormatEnum

`ImageFileChooser(ImageFormatEnum format,
File directory)`
  
          Constructs a ImageFileChooser.

`ImageFileChooser(ImageFormatEnum format,
String directory)`
  
          Constructs a ImageFileChooser.

Uses of ImageFormatEnum in org.proteinshader.gui.viewing

Constructors in org.proteinshader.gui.viewing with parameters of type ImageFormatEnum

`ScreenShot(ImageFormatEnum format,
File file)`
  
          Constructs a ScreenShot.

---


|  |  |  |
| --- | --- | --- |
| **Overview** \| **Package** \| **Class** \| **Use** \| **Tree** \| **Deprecated** \| **Index** \| **Help** | |  |
| PREV   NEXT | **FRAMES**    **NO FRAMES**     **All Classes** |  |


---

# *Copyright © 2007-2008*
